# Modelling multi-protein complexes using PELDOR distance measurements for rigid body minimisation experiments using XPLOR-NIH

**DOI:** 10.1016/j.ymeth.2014.10.028

**Published:** 2014-12

**Authors:** Colin M. Hammond, Tom Owen-Hughes, David G. Norman

**Affiliations:** a Centre for Gene Regulation and Expression, University of Dundee, Dundee DD1 5EH, UK; b Nucleic Acids Structure Research Group, University of Dundee, Dundee DD1 5EH, UK

**Keywords:** Cys, cysteine, Glu, glutamic acid, Tyr, tyrosine, Vps75, vacuolar protein sorting protein 75, PELDOR, DEER, XPLOR-NIH, MTSSLwizard, Chromatin, Vps75

## Abstract

Crystallographic and NMR approaches have provided a wealth of structural information about protein domains. However, often these domains are found as components of larger multi domain polypeptides or complexes. Orienting domains within such contexts can provide powerful new insight into their function. The combination of site specific spin labelling and Pulsed Electron Double Resonance (PELDOR) provide a means of obtaining structural measurements that can be used to generate models describing how such domains are oriented. Here we describe a pipeline for modelling the location of thio-reactive nitroxyl spin locations to engineered sties on the histone chaperone Vps75. We then use a combination of experimentally determined measurements and symmetry constraints to model the orientation in which homodimers of Vps75 associate to form homotetramers using the XPLOR-NIH platform. This provides a working example of how PELDOR measurements can be used to generate a structural model.

## 1 Introduction

To date there have been nearly 100,000 structures deposited in the protein data bank (PDB). This rich source of structural biological information is utilised by many laboratories to gain functional insights. Many structures deposited in the PDB are of individual protein domains, which are constituents of larger macromolecular complexes or proteins. Orientation of domains of known structure within such larger assemblies can often add significantly to the understanding of how different protein modules function together.

In this paper, a procedure is described which utilises Pulsed Electron Double Resonance (PELDOR) [alternatively referred to as double electron–electron resonance (DEER)] distance measurements as restraints to dock PDB structures together. This procedure can easily be extrapolated to utilise other sources of distance information, such as residue specific crosslinking information obtained from cross-linking MS/MS experiments. The protocol focusses on a molecular modelling workflow and assumes PELDOR distance measurements have been obtained, and requires access to the molecular visualisation package PyMol (http://pymol.org/) [1]. An additional plugin, MTSSLwizard [2], is required to dock the locations of spin labels onto previously determined structures. This is available at http://www.pymolwiki.org/index.php/MtsslWizard and installed by placing the python script in the appropriate directory. XPLOR-NIH [3] is then used to model spin labelled structures using experimentally obtained distance constraints. XPLOR-NIH is available at, (http://nmr.cit.nih.gov/xplor-nih/), in versions designed to run on Linux and Mac operating systems.

The working example referred to in this paper is that of a histone chaperone called Vacuolar Protein Sorting 75 (Vps75). X-ray crystallography has been used to show that Vps75 adopts homodimeric “headphone” fold conformations [4–6]. However, in solution Vps75 was recently discovered to adopt a tetrameric conformation [7]. In order to obtain insight into how two Vps75 homo-dimers were arranged within the tetrameric particle a series of PELDOR distance measurements were made at moderate salt concentrations [7]. These measurements were used to dock together two identical Vps75 dimer crystal structures as rigid bodies using the molecular modelling software XPLOR-NIH. The final model was further validated by mutagenesis experiments.

## 2 Methods

### 2.1 In silico MTSL ‘R1’ labelling of Vps75 with MTSSLwizard and formatting pdb files for XPLOR-NIH

**Fig. 1:**
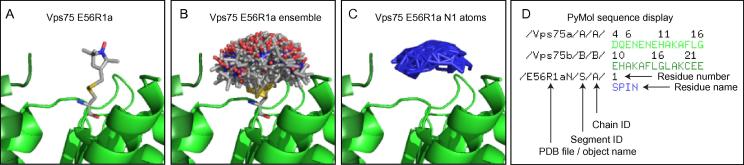
Extraction and formatting of the nitroxide nitrogen coordinates of the E56R1a spin label ensemble. (A) 1/200 conformers produced as a result of labelling E56 of Vps75 chain A with MTSL using MTSSLwizard. (B) The full spin label ensemble, “set all_states, on”, at position E56R1a. (C) The ensemble of 190 nitroxide N1 atoms extracted into the E561aN.pdb file. (D) An annotated screen from PyMol, with sequence display on, highlighting key identifiers within each PDB file which are utilised in subsequent XPLOR-NIH modelling steps.

PELDOR is an Electron Paramagnetic Resonance (EPR) experiment in which the distance between two spin labels is measured. Spin label pairs are usually incorporated into proteins by the cross-reaction of cysteine residues with a sulfhydryl reactive nitroxide radical containing compound such as S-(2,2,5,5-tetramethyl-2,5-dihydro-1H-pyrrol-3-yl)methyl methanesulfonothioate (MTSL). This spin labelled side chain is usually referred to as R1 and is prefixed by the amino acid number. For example, Vps75 E56R1 refers to Vps75 in which Glu 56 which has been mutated to Cys and cross-reacted with MTSL (Fig. 1A).

Load the PDB structure of Vps75 [4] by typing 2ZD7 into the PDB loader service plugin if available or by downloading the 2ZDZ.pdb file from http://www.rcsb.org/. To remove solvent and select the relevant chains of Vps75 for modelling purposes execute the following PyMol commands:

**Table:** 

extract Vps75a, chain a
extract Vps75b, chain b
remove HETATM
delete 2ZD7

XPLOR-NIH uses segment IDs to select different polypeptide (or other) chains within a macromolecular assembly to allow various operations to be performed on these segments in isolation. To assign new segment IDs to each chain of Vps75 perform the following commands in PyMol:

**Table:** 

alter chain A, segi=‘A’
alter chain B, segi=‘B’

Next, spin labels are introduced at the site E56R1 in Vps75 using MTSSLwizard [2]. Under the wizard menu in PyMol open the pre-installed “MTSSLwizard”. It is recommended to use the default settings of MTSSL Wizard for the initial R1 labelling of sites in PyMol. If no spin label ensemble is obtained try increasing the thoroughness or reducing the VdW restraints for the conformer search. For introducing spin labelling sites the following default settings were used:

**Table:** 

Mode: Search
Label: MTSSL
Speed: thorough search
vdW restraints: tight

To select a residue to spin label, ensure the Mouse Mode of PyMol is set to 3-Button Editing mode and click the residue of interest. This is easiest by turning on the amino acid sequence on (Display > Sequence) and scrolling through the sequence and clicking on the residue of interest. With the residue of interest selected click on “Search conformers!”. This produces an ensemble of up to 200 spin labelled R1 side chains Fig. 1B), the coordinates of which are required and their spatial relationship to the underlying protein are important for subsequent modelling steps.

Perform labelling steps for residue E56 of Vps75a duplicate the label as a new object, rename the object E56R1a. Then execute the following PyMol commands:

**Table:** 

alter E56R1a, segi=‘S’
alter E56R1a, resi=‘1’
alter E56R1a, resn=‘SPIN’

Segment IDs along with other identifiers can also be checked in PyMol when the sequence display turned on (Fig. 1D). Repeat the labelling steps for E56 of Vps75b as previously. Then execute the following PyMol commands, note the residue number has increased by 1:

**Table:** 

alter E56R1b, segi=‘S’
alter E56R1b, resi=‘2’
alter E56R1b, resn=‘SPIN’

**Table:** 

File>Save Molecule
Select Vps75a, Vps75b, E56R1a and E56R1b
Save to... multiple files
Saved state... all
OK
Save as .pdb files

Staying within the same PyMol session execute the following commands:

**Table:** 

alter segi A, segi=‘C’
alter segi B, segi=‘D’
alter segi S, segi=‘T’

Save the objects as separate pdb files.

**Table:** 

File>Save Molecule
Select Vps75a, Vps75b, E56R1a and E56R1b
Save to... multiple files
Saved state... all
OK

But this time save the files as Vps75c, Vps75d, E56R1c and E56R1d.

The coordinates of all of the nitroxide (N1) nitrogen atoms of the spin labels in the ensemble are extracted to a new file and formatted for XPLOR-NIH (Fig. 1C). Below is an example of how the N1 atoms should be extracted and formatted. This stage can be done manually using a text editor but is fairly laborious. Alternatively the pdb files generated can be modified using a Unix/Linux terminal in a more automated fashion.

PDB file character spacing (not present in files):

**Table:** 

One spin label of the ensemble in E56R1a.pdb before extraction, the required N1 atom coordinates highlighted in grey:

**Table:** 

All of the lines containing N1 atoms are extracted:

**Table:** 

Each atom within this “SPIN” residue needs to be unique and so N1 atoms are converted to N001, N002…N190. There is a limit of 190 atoms per residue in XPLOR-NIH and thus atoms above N190 are deleted. A final line with the word “END” is required for subsequent input scripts to work properly. Correct formatting of spin label ensemble for XPLOR-NIH:

**Table:** 

As mentioned the formatting of the spin label pdb files can be automated in Unix/Linux using the following two commands:

(1) cat E56R1a.pdb | grep N1 | head −190 | awk ‘{print substr($0,1,12) sprintf(“N%03d”,NR) substr($0,17)}’ > E56R1aN.pdb
(2) echo END >> E56R1aN.pdb

The newly created E56R1aN.pdb file is now correctly formatted for subsequent XPLOR-NIH molecular modelling steps. Repeat the process for the remaining spin label pdb files (E56R1b, c and d).

Particular attention should be paid to the following identifiers:

**Table:** 

13 – 16 Atom name – N001 increases incrementally to N190, the maximum number of atoms per residue in XPLOR-NIH.
18 – 21 Residue name – SPIN for each of the spin label ensembles.
23 – 26 Residue sequence number – an integer, all atoms of a single spin label ensemble have the same integer and the integer must increase by 1 for each spin ensemble eg. E56R1aN.pdb = 1 whereas E56R1bN.pdb = 2.
73 Segment id – This supersedes the pymol chain identifier at position 22 and is used for the same purpose in XPLOR-NIH
Note: Spaces between characters should not be replaced by tabs which can occur using some text editors to copy and paste columns.

Remove any lines beginning with NUMMDL, MODEL, ENDMDL or TER from the Vps75a-d.pdb files, the file should only contain lines beginning ATOM with a final line contain the word END.

**Table:** 

This formatting can also be automated in Unix/Linux using the following commands:

(1) cat Vps75a.pdb | grep ATOM > Vps75aN.pdb
(2) echo END >> Vps75aN.pdb

Repeat the process for the remaining Vps75 pdb files.

### 2.2 In silico labelling of proteins with 3,4-bis(MTSL)

The spin labelling compound 3,4-bis(MTSL) contains two thiol reactive groups and is thus capable of cross reacting with two cysteine residues that are in close proximity with one another. The benefits of this approach are twofold. One, the conformation of the spin labelled side chain is more restrained and two, the compound can be used to cross-link label two polypeptide chains. In this manner the Vps75 dimer can be singly spin-labelled by introducing a cysteine residue at position Tyr 35 which comes into close proximity with itself on the opposing monomer of the Vps75 dimer and cross-reacting the mutant protein with 3,4-bisMTSL. In this instance Vps75 Y35Rx2 denotes a Vps75 dimer that has been cross-link labelled the newly created spin labelled side chain is called Rx2. The in silico production of the conformational ensemble for the spin labelled Vps75 Y35Rx2 could not be performed using MTSSLwizard. Due to the pseudo-cyclic nature of the Rx2 side chain using a rotamer search to sample the conformational space of the spin label is not appropriate. Instead the conformational ensemble of the spin label was produced using molecular dynamics in XPLOR-NIH.

The coordinates of the N1 atoms in the spin label ensemble were then extracted and formatted as with R1 spin labelling sites. For Y35Rx2 associated with Vps75 segments A and B was assigned residue number ‘3’ and segment ID ‘S’ (see file Y35Rx2aN.pdb). While, Y35Rx2 associated with Vps75 segments C and D was assigned residue number 3 and segment ID ‘T’ (see file Y35Rx2bN.pdb).

### 2.3 Creating protein structure file for the assembly

The protein structure file (PSF) provides a full description of the macromolecular system to enable a particular force field to be applied to the system of interest. The PSF file is populated by reading in residue sequences from pdb files (as prepared in the previous sections) and using topology and parameter files to populate the properties of each atom in the system. The resultant PSF file describes how atoms in the system are connected by bonds, angles, dihedrals and improper bond angles along with other properties such as partial charges and atom masses but contains no information on the location of each atom. The location or coordinates of each atom is specified by the combined pdb file which is generated at the same time as the PSF file.

Before populating the PSF file create a new directory, the name of this directory is arbitrary. Then within this directory create two directories one called PDB and on called RES.

**Table:** 

mkdir Methods
cd Methods
mkdir RES
mkdir PDB

Within the PDB directory place the pdb files that describe the system as per Table 1. If the formatting was completed correctly as per the previous sections the residue number (character 26) and the segment ID (character 73) should match those in Table 1.

The following section describes the XPLOR-NIH script “generateQx.inp” which is shown below. After reading in topology (topallh22xedit.pro and topallhdgspin.spn) and parameter files (parallh22x.pro and parallhdg.spn) for the protein and spin labels, the PSF file for the two Vps75 dimers and associated spin labels can be populated. For the protein containing segments, the segment specific PDB file is specified after the chain command with another file toph22.pep. This allows the sequence of residues from the pdb file to be read and added to the PSF file along with information from the previously specified topology and parameter files. In the case of spin label segments S and T, notice the sequence SPIN SPIN SPIN is input after the chain command. For segment S this sequence is then populated by the E56R1aN, E56R1bN and Y35Rx2a pdb files which describe separate “SPIN” residues numbered sequentially 1, 2, 3. Segment T is populated in a similar manner. Once all segment sequences have been read in, the coordinates are read and hydrogen atoms are added. A final translation vector is used to separate the two superimposed Vps75 dimers and associated spin labels prior to writing out the final PDB and PSF files which become input files for subsequent modelling steps.

Navigate to the ‘Methods’ folder, start the XPLOR-NIH program in the Unix/Linux terminal and paste in the following statements section by section.

**Table:** 

!------------------------------------------------------------------------------------
remarks file generate/generate.inp
remarks Generate structure file and hydrogens for a protein
topology @topallh22xedit.pro
@topallhdgspin.spn
end {∗Read topology file.∗}
parameter
@parallh22x.pro {∗Read empirical potential∗}
@parallhdg.spn {∗with modifications. ∗}
nbonds {∗This statement specifies the ∗}
atomcdie shift eps=1.0 e14fac=0.4 {∗nonbonded interaction energy ∗}
cutnb=7.5 ctonnb=6.0 ctofnb=6.5 {∗options. Note the reduced ∗}
nbxmod=5 vswitch {∗nonbonding cutoff to save some∗}
end {∗CPU time. This statement ∗}
{∗overwrites the defaults in ∗}
{∗the parameter file. ∗}
end
!-------------------------------------------------------------------------------------------

**Table:** 

!--------------------------------------------------------------------------------
segment number =4 {∗Generate protein.∗}
name=“A” {∗This name has to match the ∗}
{∗four characters in columns 73∗}
{∗through 76 in the coordinate ∗}
{∗file; in XPLOR this name is ∗}
{∗referred to as SEGId. ∗}
chain
@toph22.pep
coordinates @PDB/Vps75aN.pdb
end {∗obtain the sequence. ∗}
end
!-------------------------------------------------------------------------------------------

**Table:** 

!-----------------------------------------------------------------------------------
segment number =10 {∗Generate protein.∗}
name=“B” {∗This name has to match the ∗}
{∗four characters in columns 73∗}
{∗through 76 in the coordinate ∗}
{∗file; in XPLOR this name is ∗}
{∗referred to as SEGId. ∗}
chain
@toph22.pep
coordinates @PDB/Vps75bN.pdb
end {∗obtain the sequence. ∗}
end
!--------------------------------------------------------------------------------------------

**Table:** 

!------------------------------------------------------------------------------------
segment number =4 {∗Generate protein.∗}
name=“C” {∗This name has to match the ∗}
{∗four characters in columns 73∗}
{∗through 76 in the coordinate ∗}
{∗file; in XPLOR this name is ∗}
{∗referred to as SEGId. ∗}
chain
@toph22.pep
coordinates @PDB/Vps75cN.pdb
end {∗obtain the sequence. ∗}
end
!--------------------------------------------------------------------------------------------

**Table:** 

!-----------------------------------------------------------------------------------
segment number =10 {∗Generate protein.∗}
name=“D” {∗This name has to match the ∗}
{∗four characters in columns 73∗}
{∗through 76 in the coordinate ∗}
{∗file; in XPLOR this name is ∗}
{∗referred to as SEGId. ∗}
chain
@toph22.pep
coordinates @PDB/Vps75dN.pdb
end {∗obtain the sequence. ∗}
end
!-------------------------------------------------------------------------------------------

**Table:** 

!------------------------------------------------------------------------------------
segment {∗Generate protein.∗}
name=“S”
chain
SEQUence SPIN SPIN SPIN end
end {∗obtain the sequence. ∗}
end
!------------------------------------------------------------------------------------
segment {∗Generate protein.∗}
name=“T”
chain
SEQUence SPIN SPIN SPIN end
end {∗obtain the sequence. ∗}
end
!-------------------------------------------------------------------------------------------

**Table:** 

!------------------------------------------------------------------------------------
{∗Sometimes different atom∗}
vector do (name=“O”) (name OT1) {∗names are used. ∗}
vector do (name=“OXT”) (name OT2)
vector do (name=“CD1”) (name CD and resname ile)
coordinates @PDB/Vps75aN.pdb
coordinates @PDB/Vps75bN.pdb
coordinates @PDB/Vps75cN.pdb
coordinates @PDB/Vps75dN.pdb
coordinates @PDB/E56R1aN.pdb
coordinates @PDB/E56R1bN.pdb
coordinates @PDB/E56R1cN.pdb
coordinates @PDB/E56R1dN.pdb
coordinates @PDB/Y35Rx2a.pdb
coordinates @PDB/Y35Rx2b.pdb
flags exclude vdw elec end {∗Do QUICK hydrogen building w/o∗}
{∗vdw and elec terms. ∗}
hbuild {∗This statement builds ∗}
selection=(hydrogen) {∗missing hydrogens, which are∗}
phistep=45 {∗needed for the force field. ∗}
end
delete select = (not known) end
coor translate vector (0 100 0) select = (segid A or segid B or segid S)
end
{∗The two Vps75 dimers are no longer superimposed∗}
write structure output =Vps75tet.psf end
write coor output =Vps75tet.pdb end
!-------------------------------------------------------------------------------------------

## 3 Results

### 3.1 Rigid body minimisation of PDB structures using PELDOR distance restraints

#### 3.1.1 Define interactions

If following on from the previous section, in which the starting PSF and PDB files Vps75tet.pdb and Vps75tet.psf files were created, then skip this first section of code. If starting a fresh XPLOR-NIH session then the initial parameter and topology files along with the starting PSF and PDB files will have to read in again. A short script called xprep.inp is called to do so.

**Table:** 

!-----------{∗xprep.inp∗}-------------------------------------------------------
remarks file generate/generate.inp
remarks Generate structure file and hydrogens for a protein
topology @topallh22xedit.pro
@topallhdgspin.spn
end {∗Read topology file.∗}
parameter
@parallh22x.pro {∗Read empirical potential∗}
@parallhdg.spn {∗with modifications. ∗}
nbonds {∗This statement specifies the ∗}
atom cdie shift eps=1.0 e14fac=0.4 {∗nonbonded interaction energy ∗}
cutnb=7.5 ctonnb=6.0 ctofnb=6.5 {∗options. Note the reduced ∗}
nbxmod=5 vswitch {∗nonbonding cutoff to save some∗}
end {∗CPU time. This statement ∗}
{∗overwrites the defaults in ∗}
{∗the parameter file. ∗}
end
structure @Vps75tet.psf end
coor @Vps75tet.pdb
!--------------------------------------------------------------------------------------------

The remaining sections of code are all located in the “runme.inp” script. As mentioned the starting coordinates and psf files along with topology parameter files are read in by calling the above code (using the @xprep.inp command). Next the interactions within the system that will be evaluated during energy minimisation are defined. In the case of the Vps75 tetramer, only the non-bonded interaction energies between the two Vps75 dimers (segids A + B with segids C + D) are evaluated during initial rounds of energy minimisation.

**Table:** 

@xprep.inp
param nbonds wmin .00000001 end end
!------------------------------------------------------------------------------------------
constraints interaction (all)(all) end
constraints
interaction (segid A or segid B)
(segid C or segid D)
end
!------------------------------------------------------------------------------------------

Note: energy contributions from non-bonded interactions with spin labels were ignored. As each spin label ensemble represents a population of spin label conformations evaluating the energy of non-bonded interactions with the ensemble would overestimate the specific interaction energy of a single spin label. Additionally as all spin labelled proteins used in this study were observed to tetramerise, as was the unlabelled wild type protein, any additional interaction energy contribution from the spin label was deemed insignificant.

#### 3.1.2 Restraint selection

**Fig. 3:**
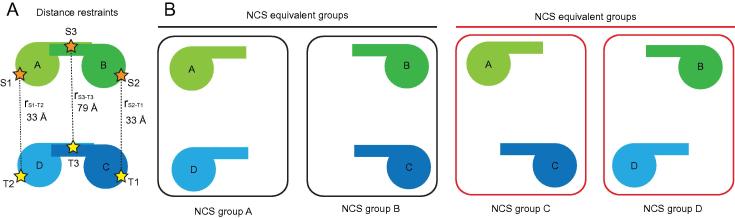
Distance (NOE) and non-crystallographic symmetry (NCS) restraints. Each Vps75 dimer is shown as a schematic labelled with the segment ID of each chain. (A) Distance restraints used to refine Vps75 tetramer structure, spin labels (depicted as stars) with associated residue identifier and segment ID. (B) Schematic representation of the two NCS equivalence groups used as restraints during rigid body minimisation. The spatial relationship between segments A and D in NCS group A should be equivalent to that between segments B and C (NCS group B). Likewise spatial relationships between segments in NCS group C should be equivalent to those in NCS group D.

Now that the interaction constraints of the system have been defined additional restraints used for the rigid body minimisation of the two Vps75 dimers into the tetrameric conformation can be defined. In this example distance measurements (highlighted in Fig. 3) obtained from PELDOR experiments were used as restraints for rigid body minimisation experiments. In addition to PELDOR distance measurements non-crystallographic symmetry restraints were utilised to maintain symmetrical interaction interfaces in the Vps75 tetramer (see Fig. 3). These distance restraints, when combined with the significant steric constraints imparted by individual Vps75 dimers, were sufficient for energy minimisation experiments to consistently converge to a single solution.

#### 3.1.3 Distance restraints in XPLOR-NIH

Following on from Section 3.1 in the XPLOR-NIH interface the PELDOR distance restraints are called in a similar manner to Nuclear Overhauser Effect (NOE) restraints obtained from NMR experiments. The only difference is the PELDOR distance measurements are much larger than typical NOE restraints. The distance restraints as depicted in Fig. 3A are input into XPLOR-NIH as follows:

**Table:** 

!------------------------------------------------------------------------------------
noe reset end
noe
nres 7000
class epr
set message=on echo=on end
{∗ distance restraints from rest.tbl file ∗}
assign (SEGID S and resid 1 and name N∗)
(SEGID T and resid 2 and name N∗) 33 2 2 {∗ E56R1 AC distance ∗}
assign (SEGID S and resid 2 and name N∗)
(SEGID T and resid 1 and name N∗) 33 2 2 {∗ E56R1 AC distance ∗}
assign (SEGID S and resid 3 and name N∗)
(SEGID T and resid 3 and name N∗) 78 0.1 0.1{∗ Y35Rx2 distance ∗}
{∗ parameters for NOE energy term ∗}
averaging epr R-3
potential epr square
sqconstant epr 1.
sqexponent epr 2
ceiling = 50.
scale epr 10
end
flags include noe end {turns on noe potential}
!------------------------------------------------------------------------------------

#### 3.1.4 Non-crystallographic symmetry considerations

As mentioned in section 3.2 the inherent symmetry in the homo-tetrameric system can also be utilised as a restraint during rigid body minimisation experiments. As shown in Fig. 3B the spatial relationship between atoms in segment A to atoms in segment D (NCS group A) is approximately equivalent to the spatial relationship between atoms in segment B and segment C (NCS group B). However, as the two chains of Vps75 are not identical the selection of each segment used to define each NCS restraint is reduced to only residues that are present within both chains of Vps75. Additionally only CA atoms were used to define each NCS restraint to account for changes in side chain orientations and slight deviations in the protein backbone. Defining the NCS restraints in this manner prevents the NCS energy from dwarfing contributions to the overall energy from other distance restraints and non-bonding interactions during energy minimisation experiments. The NCS restraints as depicted in Fig. 3B are input into XPLOR-NIH as follows:

**Table:** 

!------------------------------------------------------------------------------------
ncs restraints
init
group
equi ((segid A or segid D) and (name CA) and
(resid 10:225) and not (resid 129:136))
equi ((segid B or segid C) and (name CA) and
(resid 10:225) and not (resid 129:136))
weight-ncs=1.
sigb=1.0
end
group
equi ((segid A or segid C) and (name CA) and
(resid 10:225) and not (resid 129:136))
equi ((segid B or segid D) and (name CA) and
(resid 10:225) and not (resid 129:136))
weight-ncs=1.
sigb=1.0
end
end
flags include ncs end {turns on ncs potential}
!------------------------------------------------------------------------------------------

#### 3.1.5 Rigid body minimisation

Initially the two Vps75 dimers were orientated using the PELDOR distance restraints alone. In order to do so the nbonds repel function was set to a low value. This allows atoms to move pass each other in order to satisfy the PELDOR distance restraints.

**Table:** 

!-------{∗minimise only noe∗}-------------
flags exclude ncs end {∗turn off ncs restraints∗}
param nbonds repel = 0.000000001 end end {∗allow atoms to move past each other∗}
minimize rigid
nstep=100 drop=10.
group= (segid A or segid B or segid S)
group= (segid C or segid D or segid T)
end
write coor output=RES/noe.pdb end
!-----------------------------------------------------------------------------------------

**Fig. 4:**
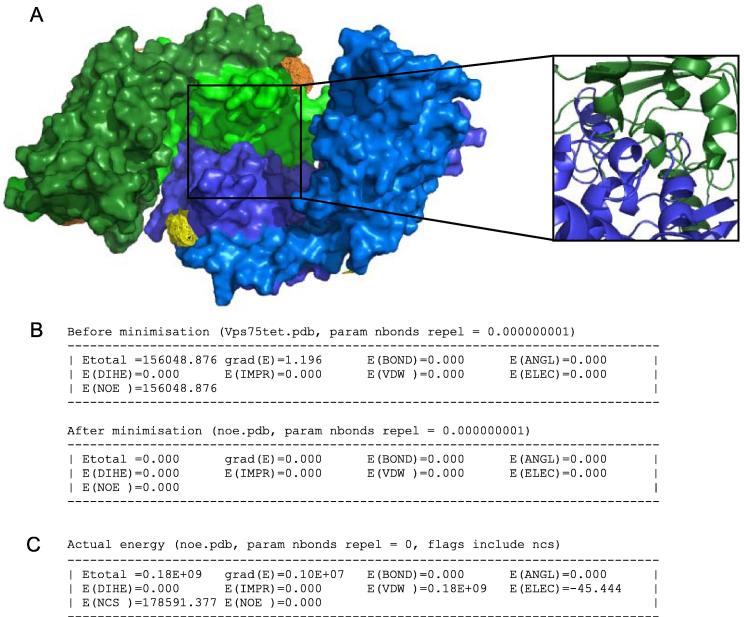
Refining a Vps75 tetramer based on PELDOR derived distance restraints in the absence of other energy terms. (A) The final coordinates of two Vps75 dimers refined by rigid body energy minimisation against PELDOR distance restraints experimentally obtained from the Vps75 tetramer. Each Vps75 dimer is differentially coloured in blue or green, with each monomer in the respective dimer shaded differentially. The region expanded demonstrates significant steric clashes between individual Vps75 dimers as a result of allowing atoms to move past each other by reducing atomic radii (nbonds = 0.000000001). (B) A comparison of the XPLOR-NIH energy terms of the starting coordinates followed by the energy terms of the refined coordinates depicted in panel A. Note the NOE energy term refines to zero demonstrating that all of the PELDOR derived distance restraints are satisfied. No other energy terms were calculated during the minimisation. (C) A calculation of the energy of the refined coordinates in panel A when other energy terms (VDW, ELEC and NCS) were calculated (nbonds = 0, resets atoms radii to default values). Note the large VDW energy term due to steric clashes indicated in panel A.

Following the first round of energy minimisation, the two dimers of Vps75 become orientated so that one globular domain of each dimer clashes with one globular domain of the opposing dimer (Fig. 4A). This orientation fully satisfies the PELDOR distance measurements input as NOE restraints in the absence of NCS, VDW and ELEC energy terms which have been turned off (Fig. 4B). However, large unfavourable energy contributions of NCS and VDW terms are apparent when the same structure is evaluated in the presence of these energy terms (Fig. 4C). Note: for each refinement step the energy of the starting coordinates are quoted along with the energy of coordinates following energy minimisation (e.g. Fig. 4B). These energy terms have been evaluated with identical parameters and interaction constraints and thus can be compared directly.

Next the NCS symmetry restraints are reintroduced and the model refined once again allowing atoms to move pass each other.

**Table:** 

!-------------{∗minimise only noe and ncs∗}--------------
flags include ncs end {∗turn on ncs restraints∗}
param nbonds repel = 0.000000001 end end {∗allow atoms to move past each other∗}
minimize rigid
nstep=200 drop=10.
group= (segid A or segid B or segid S)
group= (segid C or segid D or segid T)
end
write coor output=RES/noencs.pdb end
!-------------------------------------------------------------------------------------------

**Fig. 5:**
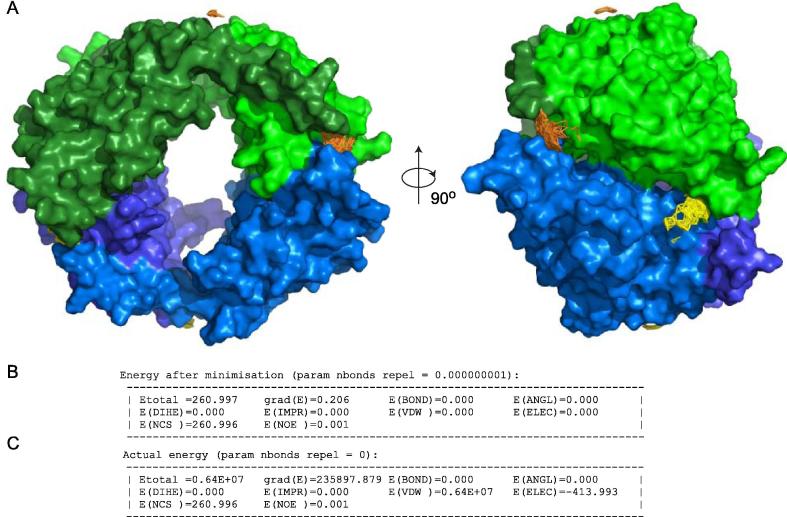
Refining a Vps75 tetramer based on PELDOR derived distance restraints and additional NCS restraints in the absence of other energy terms. (A) The final coordinates of two Vps75 dimers (as depicted in Fig. 4A) further refined by rigid body energy minimisation against NCS restraints (as depicted in Fig. 3) in addition to the PELDOR distance restraints experimentally obtained from the Vps75 tetramer. Each Vps75 dimer is differentially coloured in blue or green, with each monomer in the respective dimer shaded differentially. Due to the additional NCS symmetry restraints the two dimers of Vps75 refine into a ring-like tetramer. (B) The energy terms of the refined coordinates depicted in panel A. Note the NOE energy term refines to almost zero demonstrating that all of the PELDOR derived distance restraints are mostly satisfied. The NCS energy term refines to a reasonable level from 178,591 kcal mol^−1^ (Fig. 4C) to 261 kcal mol^−1^. The small residual NCS energy term is likely a result of intrinsic asymmetry in the Vps75 dimer. (C) A calculation of the energy of the refined coordinates in panel A when other energy terms (VDW, ELEC) were calculated (nbonds = 0, resets atoms radii to default values). Note the large VDW energy term due to steric clashes at the tetramerisation interface. Note also the favourable ELEC energy term demonstrating surface charge complementarity at the Vps75 tetramerisation interface despite the presence of steric clashes.

Refining the model with additional NCS restraints causes a re-orientation of the two Vps75 dimers which now form an alternative ring-like assembly (Fig. 5A) whilst satisfying the PELDOR distance restraints (Fig. 5B). In this orientation the earmuff domains of opposing Vps75 dimers form a smoother interface than without the NCS restraint (Fig. 4A). Although the optimised NCS and NOE energy terms are accompanied by a favourable drop in the ELEC energy term steric clashes between the Vps75 dimers persist with a large VDW energy term (Fig. 5C).

In order to reduce steric clashes between the opposing dimers of Vps75 a round of rigid body energy minimisation was performed increasing the nbonds repel function to 0.7.

**Table:** 

!---------{∗minimise noe, ncs and vdw∗}-----------
param nbonds repel = 0.7 end end
minimize rigid
nstep=100 drop=10.
group= (segid A or segid B or segid S)
group= (segid C or segid D or segid T)
end
write coor output=RES/noencsvdw.pdb end
!-------------------------------------------------------------------------------------------

This reintroduces the van der Waals energy term to the overall system energy function and as a result the proximity of atoms at the tetramerisation interface is increased (not shown) but the Vps75 tetramer retains the ring-like orientation as observed previously (Fig. 5A). Reducing steric clashes at the tetramerisation interface in such a manner improves subsequent refinement steps, where side chains are allowed to move. Note: that the VDW energy term is significantly reduced from 0.64 × 10^7^ kcal mol^−1^ (Fig. 5C) to 55 kcal mol^−1^ (param nbonds repel = 0) after this refinement step (see below) and the total energy of the system is significantly improved.

**Table:** 

Before minimisation (noencs.pdb, param nbonds repel = 0.7).
------------------------------------------------------------------------------------
| Etotal =115454.112 grad(E)=212.163 E(BOND)=0.000 E(ANGL)=0.000 |
| E(DIHE)=0.000 E(IMPR)=0.000 E(VDW)=115193.115 E(ELEC)=0.000 |
| E(NCS)=260.996 E(NOE)=0.001 |
------------------------------------------------------------------------------------
After minimisation (noencsvdw.pdb, param nbonds repel = 0.7)
------------------------------------------------------------------------------------
| Etotal =902.976 grad(E)=4.472 E(BOND)=0.000 E(ANGL)=0.000 |
| E(DIHE)=0.000 E(IMPR)=0.000 E(VDW)=68.138 E(ELEC)=0.000 |
| E(NCS)=600.602 E(NOE)=234.236 |
------------------------------------------------------------------------------------
Actual energy (noencsvdw.pdb, param nbonds repel = 0)
------------------------------------------------------------------------------------
| Etotal =674.690 grad(E)=1.125 E(BOND)=0.000 E(ANGL)=0.000 |
| E(DIHE)=0.000 E(IMPR)=0.000 E(VDW)=55.370 E(ELEC)=-215.518 |
| E(NCS)=600.602 E(NOE)=234.236 |
------------------------------------------------------------------------------------

**Table:** 

NOE energy breakdown:
++++++++++++ CLASS EPR ++++++++++++++
for this class: SCALe=10.000 AVERage=R-3 POTEntial=square-well
R<average> = 37.937 NOE = 33.00 (−2.00/+2.00) Delta = -2.937 E(NOE)= 86.259
R<average> = 37.937 NOE = 33.00 (−2.00/+2.00) Delta= -2.937 E(NOE)= 86.261
R<average> = 80.584 NOE = 78.00 (−0.10/+0.10) Delta = −2.484 E(NOE) = 61.716
NOEPRI: RMS diff. = 2.794, #(violat.> 0.0)= 3 of 3 NOEs
NOEPRI: RMS diff. class EPR = 2.794, #(viol.> 0.0)= 3 of 3 NOEs

#### 3.1.6 Rigid body minimisation coupled with internal coordinate space dynamics

**Fig. 6:**
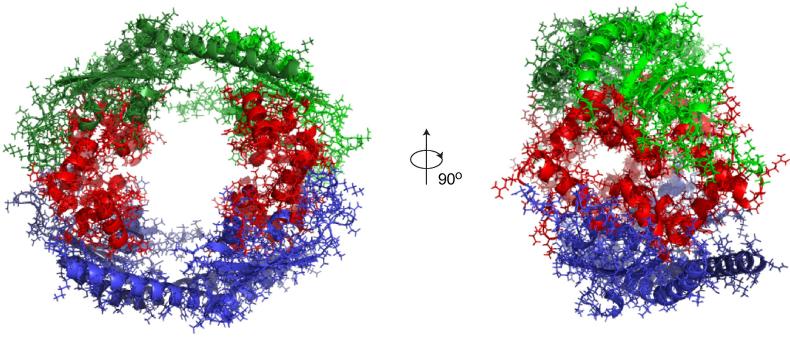
Depiction of the residues proximal to the tetramerisation interface that were allowed to move during internal coordinate space dynamics. Residues proximal to the Vps75 tetramerisation interface (57:84, 165:180, 188:194 and 211:225) are highlighted in red for both dimers. The remaining residues (1:56, 85:163,181:187 and 195:210) which are kept rigid during internal coordinate space dynamics are coloured differentially with each Vps75 dimer differentially coloured in blue or green, and each monomer in the respective dimer shaded differentially.

To further minimise the total energy of the Vps75 tetramer optimal side chain packing at the tetramerisation interface is required. To do so internal coordinate space dynamics [8,9] can be used during rigid body energy minimisation experiments. In this example internal coordinate space dynamics are utilised to allow to side chain atoms, proximal to the tetramerisation interface (see Fig. 6), to rotate around torsion angles during energy minimisation. To isolate torsion angle dynamics to side chains of residues at the tetramerisation interface, protein backbone atoms and other side chains are defined as rigid bodies. Finally, atoms in ring moieties of amino acid side chains were defined as rigid domains as torsion angle dynamics are illogical in cyclic systems. The aforementioned rigid body restraints for internal coordinate space dynamics are defined in XPLOR-NIH as follows:

**Table:** 

!----------------------{∗set up internal coordinate dynamics∗}---------------------
dynamics internal
reset
{∗ Keeping the back bone and CB atoms rigid allowing all other side chain
atoms to move∗}
group (segid S or ((segid A or segid B) and
(name ca or name c or name n or name or
or name hn or name ha or name ha#)))
group (segid T or ((segid C or segid D) and
(name ca or name c or name n or name o
or name hn or name ha or name ha#)))
{∗ Allow the side chains of residues at the tetramerisation interface to
move, keep the following selections rigid ∗}
group ((segid A or segid B) and (resid 1:56 or (resid 85:163) or
(resid 181:187) or (resid 195:210)))
group ((segid C or segid D) and (resid 1:56 or (resid 85:163) or
(resid 181:187) or (resid 195:210)))
{∗ group together rigid ring atoms ∗}
set message off echo off end
for $seg in (A B C D) loop m1
evaluate ($res = 10)
while ($res <225) loop m2
group (segid $seg and (resname PHE) and (resid $res) and (name CG or name CD1
or name CD2 or name CE1 or name CE2 or name CZ))
group (segid $seg and (resname HIS) and (resid $res) and (name CG or name ND1
or name CD2 or name CE1 or name NE2))
group (segid $seg and (resname TYR) and (resid $res) and (name CG or name CD1
or name CD2 or name CE1 or name CE2 or name CZ))
group (segid $seg and (resname TRP) and (resid $res) and (name CG or name CD1
or name CD2 or name NE1 or name CE2 or name CE3 or name CZ2 or name
CZ3 or name CH2))
group (segid $seg and (resname PRO) and (resid $res))
evaluate ($res = $res + 1)
end loop m2
end loop m1
set message on echo on end
auto torsion
maxe 1000
end
{∗This may take a while∗}
!-------------------------------------------------------------------------------------------

Prior steps of energy minimisation utilise each Vps75 dimer as a fully rigid body, which can move with respect to one another but are not altered in structure internally. Thus only evaluating the energy of the inter-dimer interaction is necessary during refinement. However, maintaining this interaction constraint whilst allowing torsion angle dynamics for side chains at the tetramerisation interface gives no energy penalty to intra-dimer side chain clashes whilst minimising the energy of inter-dimer side chain interactions. Thus the interaction energy of intra-dimer side chains is also evaluated during the final round of internal coordinate energy minimisation. Note: intra-dimer electrostatics energy terms did not contribute to the overall energy to prevent this term from dominating the minimisation procedure. The interaction constraints are specified as follows:

**Table:** 

!----{∗evaluate the energy between residues at the tetramerisation interface∗}----
constraints interaction (all) (all) end
constraints
interaction (segid A or segid B) (segid C or segid D) weights ∗ 1. end
interaction (segid A and (resid 57:84 or resid 165:180 or resid 188:194 or resid 211:225))
(segid A and (resid 57:84 or resid 165:180 or resid 188:194 or resid
211:225))
weights ∗ 1. elec 0 end
interaction (segid B and (resid 57:84 or resid 165:180 or resid 188:194 or resid
211:227))
(segid B and (resid 57:84 or resid 165:180 or resid 188:194 or resid
211:227))
weights ∗ 1. elec 0 end
interaction (segid C and (resid 57:84 or resid 165:180 or resid 188:194 or resid
211:225))
(segid C and (resid 57:84 or resid 165:180 or resid 188:194 or resid
211:225))
weights ∗ 1. elec 0 end
interaction (segid D and (resid 57:84 or resid 165:180 or resid 188:194 or resid
211:227))
(segid D and (resid 57:84 or resid 165:180 or resid 188:194 or resid
211:227))
weights ∗ 1. elec 0 end
end
!--------------------------------------------------------------------------------------------

After defining the rigid restraints and interaction constraints of the system, a round of internal coordinate space dynamics energy minimisation was performed. Note: the NCS and NOE restraints used in the preceding energy minimisation experiment were maintained during this round of energy minimisation and the nbonds repel function was reset to default.

**Table:** 

!--------------{∗minimise energy with internal coordinate dynamics∗}---------------
param nbonds repel = 0 end end
dynamics internal
itype=powell
nstep=2000
depred=1
end
write coor output=RES/noencsvdwdynint.pdb end
!--------------------------------------------------------------------------------------------

**Fig. 7:**
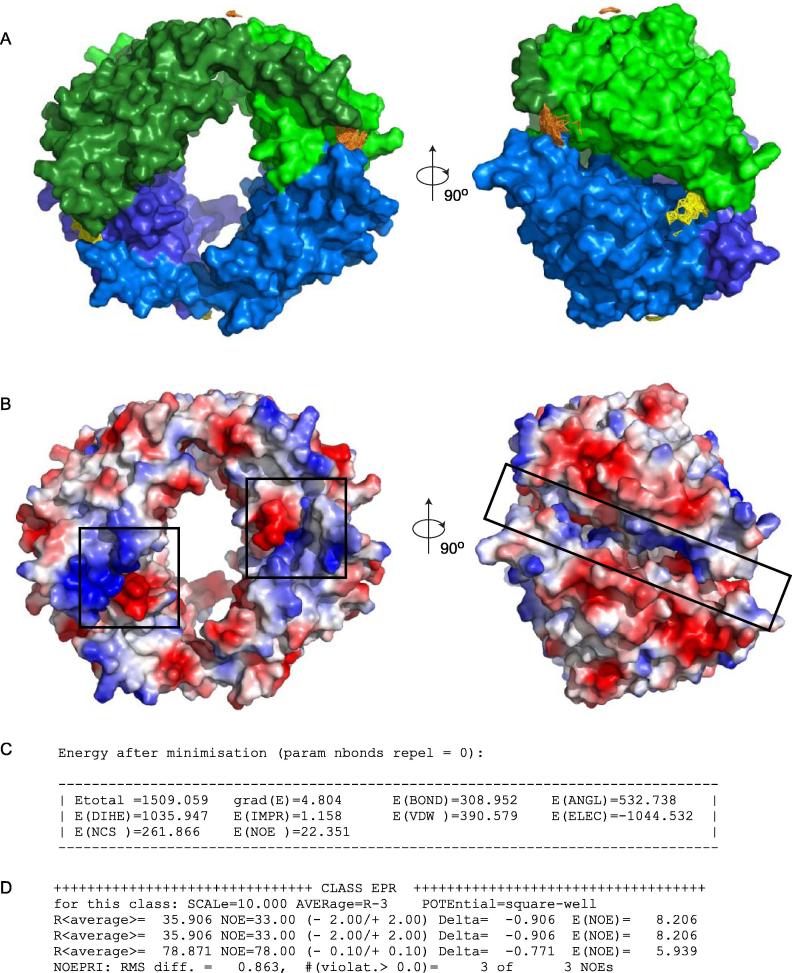
Optimising the packing of side chains at the tetramerisation interface using internal coordinate space dynamics. (A) The final coordinates of two Vps75 dimers (as depicted in Fig. 5A) further refined in two steps first by rigid body energy minimisation with NCS restraints, PELDOR distance restraints and other energy terms (not shown) and then in a subsequent refinement step with additional internal coordinate space dynamics (final coordinates depicted). Each Vps75 dimer is differentially coloured in blue or green, with each monomer in the respective dimer shaded differentially. The Vps75 tetramer maintains its ring-like appearance but with optimised packing of side chains at the tetramerisation interface. (B) Regions of surface charge complementarity at the tetramerisation interface are highlighted in black boxes, areas of positive (blue) and negative (red) charge coloured differentially. (C) The energy terms of the refined coordinates depicted in panel A. The NCS energy term remains approximately constant (compared to Fig. 5C) while the VDW energy term is reduced from 6.4 × 10^8^ kcal mol^−1^ (Fig. 5C) to 391 kcal mol^−1^ with a further reduction in the ELEC energy term from −414 kcal mol^−1^ (Fig. 5C) to −1045 kcal mol^−1^. The bulk of the DIHE, IMPR, BOND and ANGL energy terms (1879 kcal mol^−1^ total) are inherited from the crystallographically determined starting coordinates (Fig. 2) which contribute 1861 kcal mol^−1^. (D) Distances (in angstroms) between spin label ensembles in the final model (R<average>) verses experimentally determined PELDOR distance restraints (NOE). All experimentally determined distances restraints are satisfied with in 3 Å in the final model.

Following refinement with internal coordinate space dynamics the Vps75 tetramer retains the familiar ring-like orientation (Fig. 7A) observed in previous rounds of energy minimisation (Fig. 5A). Further improvements in the electrostatic surface charge complementarity are observed both in the structure (Fig. 7B) and in the final energy function (Fig. 7C). The model fits the experimentally determined distance restraints well with each restraint fit within a 3 Å deviation (Fig. 7D). Although the overall energy of the system is positive this is largely due to a combined contribution of 1879 kcal mol^−1^ from the DIHE, IMPR, BOND and ANGL energy terms. The bulk of which is inherited from the crystallographically determined starting coordinates, which when evaluated with the same interaction constraints have a combined energy for these terms of 1861 kcal mol^−1^ (not shown).

## 4 Discussion

Here we have reported a simple yet efficient molecular modelling pipeline to produce molecular models of protein–protein or domain–domain interactions. Such a workflow is well suited PELDOR distance measurements but could equally be extrapolated to utilise distance information obtained from crosslinking MS/MS type experiments. Initially the location of nitroxide radical spin labels were modelled using MTSSLwizard, this process could also be included into the XPLOR-NIH workflow using molecular dynamics. However the simplicity of the PyMol-MTSSLwizard approach works well for R1 labelling of proteins. Additionally MMM [10] or PRONOX [11] programs can be used to model the spin label ensemble. The ensemble of spin label nitrogen atom positions extracted is fixed relative to the coordinates of the underlying protein backbone for subsequent energy minimisation experiments. This approach allows the conformational dynamics of the spin label to be mimicked during energy minimisation of distance restraints between spin labels whilst preventing the spin label adopting a preferred conformation in order to satisfy the restraint. Subsequent rigid body minimisation experiments were performed using XPLOR-NIH to dock the two dimers of Vps75 together using PELDOR distance restraints. The flexibility of the XPLOR-NIH language allows a wide variety of additional restraints, such as the NCS restraints used here, to be utilised in a manner that is flexible to the user’s needs.

It is important to bear in mind that the structure of the Vps75 tetramer was refined with rigid body minimisation, the inherent assumption of which is that the internal structure of the Vps75 dimer does not change upon tetramerisation. Additionally the sparse distance constraints provided from PEDLOR experiments are not sufficient to accurately predict side chain conformations at the tetramerisation interface. However a better fit to the PELDOR distance measurements was obtained by allowing some flexibility of side chains at the tetramerisation interface. Importantly, the model presented here was further validated experimentally. Mutagenesis of residues at the tetramerisation interface identified charge reversal point mutations which significantly affected the ability of Vps75 to tetramerise - as assayed by SEC-MALS [7]. A cysteine mutant was identified that could cross-link to itself on the opposing dimer across the tetramerisation interface thus trapping Vps75 in a tetrameric conformation. Finally the model provided a good fit to Small Angle X-ray scattering data for the Vps75 tetramer [7].

## 5 Conclusions

Defining the structure of macromolecular complexes can be a challenging task for various reasons. When working with systems in which parts of the macromolecular complex are known, PELDOR distance measurements can be used to dock the component parts together. Here we describe a molecular modelling pipeline to utilise PELDOR distance restraints during rigid body minimisation experiments to dock proteins together in XPLOR-NIH. By providing a working example that is easy to follow we hope to encourage the application of this workflow to other biological problems.

## Figures and Tables

**Fig. 2:**
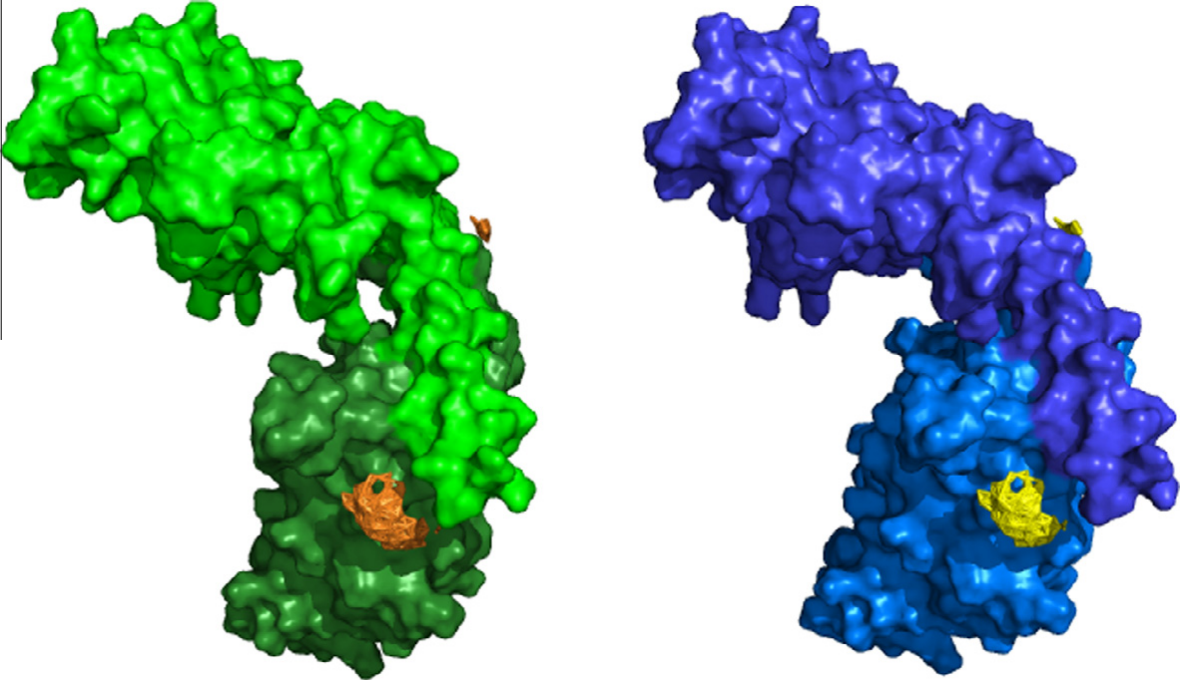
Starting coordinates of the two Vps75 dimers (green and blue) with associated nitroxide nitrogen atoms of spin label ensembles (orange and yellow) as per Vps75tet.pdb.

**Table 1:** List of PDB files required for creating the starting PDB and PSF files for molecular modelling with XPLOR-NIH. Segment ID and residue numbers are noted for each file which can be checked for consistency as deviations from values quoted above may affect subsequent steps in the protocol.

File	Segment ID	Residue sequence	
		Start	End
Vps75aN.pdb	A	4	225
Vps75bN.pdb	B	10	227
Vps75cN.pdb	C	4	225
Vps75dN.pdb	D	10	227
E56R1aN.pdb	S	1	1
E56R1bN.pdb	S	2	2
Y35Rx2a.pdb	S	3	3
E56R1cN.pdb	T	1	1
E56R1dN.pdb	T	2	2
Y35Rxb.pdb	T	3	3

## Appendix A Supplementary data

Supplementary data 1 This file contains supplementary data.

Supplementary data 2 This file contains supplementary data.
