# Supplementary material for: Modelling multi-protein complexes using PELDOR distance measurements for rigid body minimisation experiments using XPLOR-NIH
Source: Methods. 2014 Dec;70(2-3):139–53. doi: 10.1016/j.ymeth.2014.10.028 (PMC4274318; doi:10.1016/j.ymeth.2014.10.028)
Supplement: Supplementary data 1 — This file contains supplementary data. [file mmc1.docx]

Index of supplementary files provided as a compressed directory.

PDB files used to generate PSF files.

E56R1a.pdb

E56R1aN.pdb

E56R1b.pdb

E56R1bN.pdb

E56R1c.pdb

E56R1cN.pdb

E56R1d.pdb

E56R1dN.pdb

Vps75a.pdb

Vps75aN.pdb

Vps75b.pdb

Vps75bN.pdb

Vps75c.pdb

Vps75cN.pdb

Vps75d.pdb

Vps75dN.pdb

Y35Rx2a.pdb

Y35Rx2b.pdb

Results files (RES)

energy_final.out

noe.out

noe.outx

noe.pdb

noencs.pdb

noencsvdw.pdb

noencsvdwdynint.pdb

Scripts

generateQx

runme

xprep

Miscellaneous

paralh22x

paralhdg.spn

rest.tbl

topalh22xedit

topalhdgspin.spn

toph22.pep

vps75tet.pdb

vps75tet
